# Development and evaluation of a multimodal feature-based predictive model for radiotherapy-induced oral mucositis in nasopharyngeal carcinoma

**DOI:** 10.1371/journal.pone.0346251

**Published:** 2026-04-09

**Authors:** Ling Li, Linke Li, Ruifeng Guo, Shiting Fang, Ke Wang, Ge Yuan, Danxian Jiang, Jing Huang

**Affiliations:** 1 Department of Radiotherapy, Affiliated Hospital of Guangdong Medical University, Zhanjiang, Guangdong, China; 2 School of Medical Imaging, Laboratory and Rehabilitation, Xiangnan University, Chenzhou, Hunan, China; 3 Department of Head and Neck Oncology, Affiliated Hospital of Guangdong Medical University, Zhanjiang, Guangdong, China; University of Pisa, ITALY

## Abstract

**Background:**

Accurate prediction of radiation-induced oral mucositis is crucial for personalized treatment in head and neck cancer. However, developing robust predictive models utilizing high-dimensional multimodal data (CT imaging, dose distribution, and clinical features) remains challenging, particularly in cohorts with limited sample sizes.

**Objective:**

This study aimed to rigorously evaluate and compare the multi-class predictive performance of traditional machine learning algorithms and deep learning architectures under a small-cohort setting.

**Methods:**

Multimodal data from 108 patients were collected. A comprehensive evaluation framework incorporating nine traditional machine learning algorithms and two deep learning models (a dimensionality-reduced 1D-CNN and a multimodal 3D-CNN) was established. To ensure robust evaluation, a stratified 5-fold cross-validation was employed. Model performance was comprehensively quantified using mean ± standard deviation (SD) across multiple metrics, including the Area Under the Curve (AUC), accuracy, and Matthews Correlation Coefficient (MCC).

**Results:**

Inter-rater reliability for RIOM grading was excellent (Cohen’s kappa = 0.82, 95% CI: 0.73–0.91). Among traditional machine learning approaches, the Extra Trees (ET) algorithm achieved the highest discriminative capacity (AUC: 0.956 ± 0.046), while Logistic Regression (LR) demonstrated optimal overall accuracy (0.832 ± 0.155) and stability. Regarding deep learning, the lightweight 1D-CNN utilizing fused low-dimensional features exhibited highly competitive and robust performance (AUC: 0.900 ± 0.072; Accuracy: 0.732 ± 0.140). In stark contrast, the high-dimensional multimodal 3D-CNN suffered from severe overfitting and mode collapse phenomenon, yielding significantly inferior results (AUC: 0.568 ± 0.090; MCC: −0.025 ± 0.031).

**Conclusions:**

For small-cohort radiomics and dosimetric analyses, ensemble learning models (e.g., ET) and appropriately regularized linear models (e.g., LR) remain highly effective. While deep learning holds promise, high-dimensional architectures like 3D-CNNs are highly susceptible to mode collapse without massive datasets. Instead, employing feature dimensionality reduction combined with lightweight networks (1D-CNN) is a vastly superior strategy to extract reliable predictive patterns from limited clinical data.

## Introduction

Nasopharyngeal carcinoma (NPC), due to its anatomical proximity to critical structures, is primarily treated with radiotherapy (RT) and chemotherapy. Advances in radiotherapy techniques have been shown to improve locoregional control. Except for early-stage disease (T1–2N0), concurrent chemoradiotherapy has become the standard treatment regimen [1,2]. However, these treatments inevitably damage healthy tissues and lead to toxic side effects. Radiation-induced oral mucositis (RIOM) is the most common acute toxicity. A meta-analysis by Li et al. [3] reported that 99% of NPC patients developed RIOM after RT, with 52% experiencing severe RIOM. The pain and discomfort caused by RIOM significantly impair patients’ quality of life and treatment compliance, and in some cases result in unplanned hospitalizations, treatment interruptions, or chemotherapy dose reductions, thereby compromising overall therapeutic outcomes [4,5]. Therefore, accurately identifying high-risk patients for RIOM prior to radiotherapy and implementing individualized preventive and interventional strategies has become a critical issue in the management of NPC.

Previous studies have shown that the occurrence of RIOM is associated with multiple factors, including radiation dose [6], target volume [7,8], concurrent chemotherapy, patients’ baseline metabolic status (e.g., a low body mass index[BMI]), smoking [9], diabetes, and other clinical variables. In addition, different planning parameters (e.g., dose–volume histogram [DVH] metrics) and target delineation strategies can significantly affect mucosal dose exposure and the severity of toxicity [7]. However, conventional risk assessment methods are often based on single-variable linear analyses, which fail to capture nonlinear and high-dimensional interactions among diverse factors. As a result, their predictive accuracy and individualized adaptability remain limited.

In recent years, artificial intelligence (AI), particularly machine learning and deep learning methods, has been widely applied to the prediction of treatment-related toxicities in oncology. AI models can automatically learn latent structures and high-dimensional features from multimodal data, offering the capability to capture complex nonlinear relationships. Previous studies have reported promising results in this domain, demonstrating that machine learning can achieve high predictive accuracy for radiation-induced toxicities such as pneumonitis, skin injury, and brain injury [8]. Furthermore, the integration of AI and radiomics offers a unique opportunity to develop dedicated imaging biobanks, which can significantly aid in personalized patient care and clinical decision-making within the context of modern precision medicine [10]. However, most existing studies remain limited to a single modality (e.g., DVH parameters, clinical information, or radiomics features) or rely on a single modeling approach, with model generalizability and stability yet to be improved.

This study focuses on predicting RIOM in patients with nasopharyngeal carcinoma undergoing radiotherapy. We propose to construct a multimodal feature set that integrates pretreatment CT images, dose distribution data, DVH parameters, and baseline clinical information. Multiple machine learning algorithms (e.g., Light Gradient Boosting Machine [LightGBM], Xtreme Gradient Boosting [XGBoost], and random forests) as well as deep learning methods (One-Dimensional Convolutional Neural Network [1D-CNN] and Three-Dimensional Convolutional Neural Network [3D-CNN]) will be employed to develop predictive models, and their performance will be systematically compared. The aim of this research is to explore efficient, interpretable, and clinically applicable predictive strategies, thereby providing a theoretical basis and technical support for individualized risk management of radiotherapy-induced toxicities.

## Materials and methods

### Patients

This retrospective study included 108 patients with pathologically confirmed NPC who completed radiotherapy and follow-up at the Radiotherapy Center of the Affiliated Hospital of Guangdong Medical University between 1 January 2022 and 31 December 2023. The sample size was estimated using PASS 15.0 software. Based on the primary outcome (RIOM Grade ≥2 vs. < 2), with a significance level (α) of 0.05, a power (β) of 0.2, and an expected effect size of 0.8, the minimum required sample size was calculated to be 92. Therefore, the final enrollment of 108 patients met the statistical requirements for valid analysis. During data collection, the authors had access to patient identifiers for clinical follow-up purposes; however, strict data protection measures were implemented as detailed below.

Ethics Statement: This study was conducted in accordance with the protocols approved by the Clinical Research Ethics Committee of the Affiliated Hospital of Guangdong Medical University. The initial patient enrollment and collection of oral mucositis symptoms were performed under approval No. PJKT2022-002-02, for which written informed consent was obtained from all participants. The subsequent predictive modeling and data analysis were further approved under No. PJKT2025−201. All data were fully anonymized and de-identified prior to analysis to ensure patient confidentiality. The anonymized datasets supporting the findings of this study, including radiomic features, dosiomic features, clinical characteristics, and DVH parameters, are available as Supporting Information (S1–S4 Data).

Inclusion criteria were as follows: (1).Pathologically confirmed diagnosis of NPC and scheduled to undergo radiotherapy; (2).Age ≥ 18 years; (3).Availability of complete pretreatment CT images, radiotherapy planning data, and dose distribution files; (4).Oral mucositis graded according to standardized RTOG criteria during radiotherapy; (5).Provision of informed consent for the use of de-identified clinical data for research purposes. Exclusion criteria were as follows: (1) presence of significant oral mucosal ulceration or other oral diseases before radiotherapy that could affect evaluation; (2) prior history of head and neck cancer requiring re-irradiation; (3) radiotherapy interruption exceeding 7 consecutive days; (4) incomplete or poor-quality imaging or planning data; and (5) loss to follow-up or transfer to another hospital resulting in incomplete clinical records. Table 1 outlines a summary of the clinical characteristics of the patients included in this study.

**Table 1:** Summary of patients’ characteristics.

Patient variable	Number (%)	Patient variable	Number (%)
**Sex**		**N stage**	
**Male**	82(75.92%)	**N0**	11(10.19%)
**Female**	26(24.08%)	**N1**	45(41.67%)
**Age in years Median:**	52(32-73)	**N2**	29(26.85%)
**Tobacco use**	54(50%)	**N3**	23(21.30%)
**Oral mucositis 0**	0(0%)	**M stage**	
**Oral mucositis 1**	24(22.22%)	**M0**	105(97.22%)
**Oral mucositis 2**	57(52.78%)	**M1**	3(2.78%)
**Oral mucositis 3**	27(25%)	**Treatment**	
**Oral mucositis 4**	0(0%)	**IC+CCRT**	75(69.44%)
**T stage**		**(IO+)IC+CCRT**	22(20.37%)
**T1**	7(6.48%)	**IC + RT**	2(1.85%)
**T2**	6(5.56%)	**CCRT**	6(5.56%)
**T3**	61(56.48%)	**RT**	3(2.78%)
**T4**	34(31.48%)		

### Radiotherapy protocol and toxicity assessment

Oral cavity inspections and mucositis grading were jointly carried out by an associate chief physician and physicians who had received formal training. Toxicity assessments were performed at three time points: prior to the initiation of radiotherapy (baseline), once per week during the treatment course, and at an 8-week follow-up after completion of radiotherapy.

The severity of oral mucositis was classified according to the National Cancer Institute Common Terminology Criteria for Adverse Events (NCI-CTCAE, version 5.0), with grading primarily based on pain intensity and the patient’s ability to maintain oral intake. Grade 1: Mild or asymptomatic, no medical intervention required; Grade 2: Moderate pain, dietary adjustment needed but oral intake maintained; Grade 3: Severe pain with significant impairment of oral intake; Grade 4: Life-threatening consequences requiring urgent medical intervention.For each patient, the maximum toxicity grade observed during the evaluation period was recorded and used as the study endpoint.

### Data collection, ROI delineation, and feature processing

**Fig 1:**
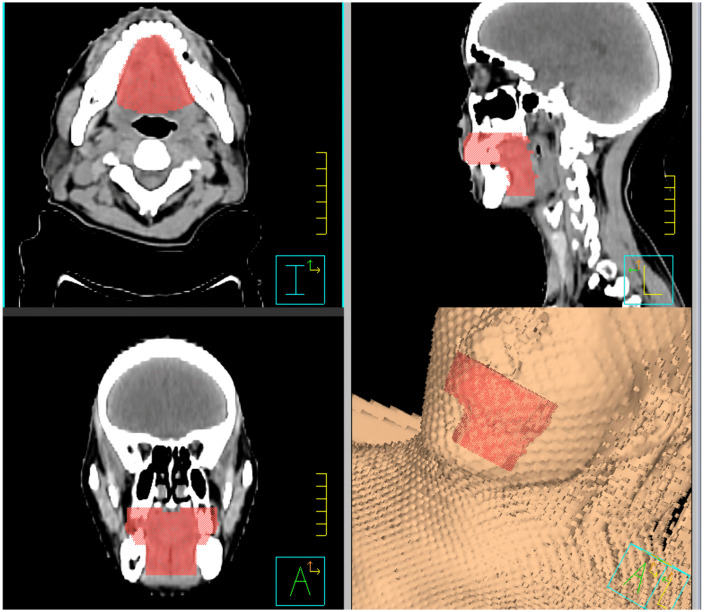
Manual segmentation of the entire oral cavity on CT images from a representative patient.

The oral cavity was contoured on the planning CT using the Pinnacle treatment planning system by two senior radiation oncologists (with 22 and 14 years of clinical experience in head and neck radiation oncology, respectively), and the contours were cross-checked for consistency (Fig 1). The delineation was performed according to the anatomic boundaries described by Bhide et al. [11], with the hard palate as the superior border, the floor of the mouth as the inferior border, the buccal mucosa surrounding the teeth as the anterior border, and the posterior tongue surface together with the uvula as the posterior border.

The dataset comprised four categories of information: CT Images: Radiomic features of the oral cavity were extracted using PyRadiomics, including shape descriptors, first-order statistics, and textural features derived from the gray-level co-occurrence matrix (GLCM), gray-level dependence matrix (GLDM), gray-level run-length matrix (GLRLM), gray-level size zone matrix (GLSZM), and neighborhood gray-tone difference matrix (NGTDM).

Dose Distribution Maps: Dosimetric features were obtained from RT Plan and Dose files, encompassing first-order statistics, dose-texture characteristics, and geometric descriptors of the dose distribution.

DVH Parameters: Dose–volume histogram metrics for the oral cavity were collected, including maximum dose (D_max_), minimum dose (D_min_), mean dose (D_mean_), V_5_–V_70_ (in 5 Gy increments), and D_5_–D_100_ (in 5% increments). Here, V_x_ (%) represents the percentage of the organ volume receiving at least x Gy, whereas D_x_ (Gy) denotes the minimum dose received by x% of the organ volume.

Clinical Characteristics: Demographic and treatment-related factors were included, such as sex, age, TNM stage, treatment modality (immunotherapy [Io], induction chemotherapy [IC], concurrent chemoradiotherapy [CCRT]), diabetes, and smoking history. Categorical variables were encoded using one-hot encoding.

Data preprocessing was conducted as follows: CT and dose images were normalized to a 0–1 intensity range. All extracted quantitative features were standardized using Z-score normalization with the StandardScaler function. Missing values were imputed with the median of each feature column. For categorical clinical variables, one-hot encoding was applied.

The dataset was randomly split into training and testing cohorts in an 80:20 ratio. Feature selection for radiomic, dosiomic, DVH, and clinical datasets was performed using the minimum redundancy maximum relevance (mRMR) algorithm, which ranks features based on information gain while minimizing redundancy. For multimodal fusion, radiomic, dosiomic, DVH, and clinical features were first normalized, then reduced in dimensionality using principal component analysis (PCA). The resulting feature sets were combined and used for model training and evaluation.

### Construction and validation of RIOM predictive models

**Fig 2:**
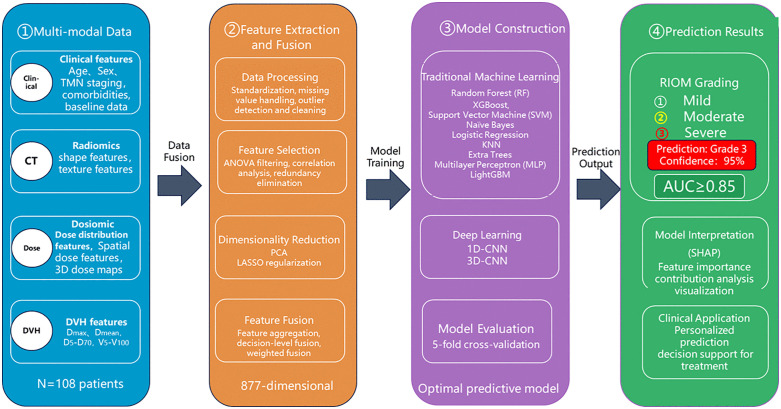
Four-Step Core Workflow Diagram.

The overall workflow of this study is illustrated in Fig 2. Two categories of predictive models were developed. Conventional Machine Learning Models (9 types): multilayer perceptron (MLP), random forest (RF), extremely randomized trees (ExtraTrees), support vector machine (SVM), k-nearest neighbors (KNN), LightGBM, XGBoost, logistic regression (LR), and naïve Bayes (NB). Deep Learning Models: 1D-CNN and 3D-CNN, with voxel-level CT and dose maps directly used as input.

Model training was performed using five-fold cross-validation. Performance was evaluated using multiple metrics, including accuracy, sensitivity, specificity, precision, F1 score, Matthews correlation coefficient (MCC), and area under the receiver operating characteristic curve (AUC, one-vs-rest). Confusion matrices and ROC curves were generated for comprehensive model comparison.

The evaluation metrics were defined as follows:


$$\text{Accuracy}=\frac{\text{TP}+\text{TN}}{\text{TP}+\text{FP}+\text{TN}+\text{FN}}$$ (1)

$$\text{Sensitivity}=\frac{\text{TP}}{\text{TP}+\text{FN}}$$ (2)

$$\text{Specificity }=\frac{\text{TN}}{\text{TN}+\text{FP}}$$ (3)

$$\text{Precision}=\frac{\text{TP}}{\text{TP}+\text{FP}}$$ (4)

$$\text{F1}-\text{ measure}=\text{2}\times \frac{\text{Precision }\times \text{Sensitivity}}{\text{Precision }+\text{Sensitivity}}$$ (5)

$$\text{MCC}=\frac{\text{TP}\times \text{TN}-\text{FP}\times \text{FN}}{\sqrt{\left(\text{TP}+\text{FP})(\text{TP}+\text{FN})(\text{TN}+\text{FP})(\text{TN}+\text{FN}\right)}}$$ (6)

where: TP: True Positive. FP: False Positive. TN: True Negative, and FN: False Negative.

### Model interpretability and modality contribution analysis

To address the “black box” nature of machine learning models and facilitate clinical adoption, we employed the SHapley Additive exPlanations (SHAP) framework to interpret the decision-making process of the optimal predictive model identified from the comparative analysis.

We conducted interpretability analysis at two levels:

(1) Feature-Level Interpretation: SHAP summary plots were generated to visualize the global importance rankings of the top features and their directional impact on toxicity grading.
(2) Modality-Level Contribution Analysis: To quantify the reliance of the model on different data sources, we performed an aggregated importance analysis. First, the mean absolute SHAP value was calculated for each individual feature. Next, these values were summed according to their respective modalities (Radiomics, Dosiomics, and Clinical). Finally, the relative contribution of each modality was derived by normalizing these sums to a percentage of the total feature importance (i.e., the sum of importances across all modalities). This approach provided a quantitative measure of the weight the model assigns to each information type.

### Model evaluation and clinical utility

To further assess the clinical utility and calibration of the optimal model, calibration curves and decision curve analysis (DCA) were performed. The calibration curve was used to visualize the consistency between the predicted risk and actual observed outcomes, quantified by the Brier score. DCA was employed to determine the clinical net benefit of the model-based intervention across different threshold probabilities.

## Results

### Patient characteristics

The clinical characteristics of the study cohort are summarized in Table 1. A total of 108 patients with nasopharyngeal carcinoma were included, with a median age of 52 years (range, 32–73 years). Among them, 82 patients (75.92%) were male and 26 (24.08%) were female. Regarding the severity of radiation-induced oral mucositis (RIOM), 24 patients (22.22%) experienced Grade 1, 57 patients (52.78%) experienced Grade 2, and 27 patients (25.00%) experienced Grade 3, while no patients developed Grade 4 RIOM.

### Performance of conventional machine learning models

Table 2 presents the performance of nine conventional machine learning models in predicting RIOM with all evaluation metrics—including accuracy, sensitivity, specificity, precision, F1 score, Matthews correlation coefficient (MCC), and area under the receiver operating characteristic curve (AUC, one-vs-rest)—reported as mean ± standard deviation (SD).

**Table 2:** Performance Comparison of Nine Machine Learning Models (5-Fold Cross-Validation, Mean ± SD).

Model	Accuracy	Sensitivity	Specificity	Precision	F1 Score	MCC	AUC (OvR)
**MLP**	0.638 ± 0.091	0.573 ± 0.136	0.774 ± 0.066	0.609 ± 0.151	0.576 ± 0.155	0.369 ± 0.181	0.771 ± 0.092
**ET**	0.759 ± 0.019	0.658 ± 0.031	0.830 ± 0.014	0.896 ± 0.008	0.700 ± 0.039	0.619 ± 0.028	0.956 ± 0.046
**RF**	0.684 ± 0.064	0.559 ± 0.111	0.778 ± 0.052	0.798 ± 0.153	0.573 ± 0.128	0.481 ± 0.111	0.940 ± 0.033
**SVM**	0.536 ± 0.128	0.495 ± 0.111	0.731 ± 0.072	0.551 ± 0.182	0.485 ± 0.108	0.238 ± 0.226	0.727 ± 0.127
**KNN**	0.463 ± 0.066	0.341 ± 0.060	0.661 ± 0.037	0.300 ± 0.118	0.301 ± 0.085	−0.020 ± 0.152	0.556 ± 0.091
**LightGBM**	0.758 ± 0.095	0.710 ± 0.146	0.846 ± 0.072	0.768 ± 0.104	0.725 ± 0.137	0.581 ± 0.195	0.901 ± 0.039
**XGBoost**	0.694 ± 0.129	0.639 ± 0.148	0.806 ± 0.081	0.703 ± 0.147	0.659 ± 0.152	0.467 ± 0.237	0.857 ± 0.051
**LR**	0.832 ± 0.155	0.804 ± 0.196	0.894 ± 0.104	0.858 ± 0.131	0.810 ± 0.191	0.710 ± 0.288	0.930 ± 0.071
**NB**	0.686 ± 0.116	0.640 ± 0.098	0.806 ± 0.058	0.734 ± 0.099	0.669 ± 0.098	0.473 ± 0.182	0.826 ± 0.099

Abbreviations: MLP, Multilayer Perceptron; ET, Extremely Randomized Trees; RF, Random Forest; SVM, Support Vector Machine; KNN, K-Nearest Neighbors; LR, Logistic Regression; NB, Naïve Bayes.

Overall, ensemble learning methods demonstrated superior predictive stability. The Extra Trees (ET) model achieved the highest discriminative performance with an AUC of 0.956 ± 0.046 and minimal variability (accuracy SD = 0.019). To assess statistical significance, paired t-tests were performed on fold-wise AUC values. The results indicated that the AUC of the ET model was significantly higher than that of XGBoost (P = 0.0178), Multilayer Perceptron (*P* = 0.0220), Support Vector Machine (*P* = 0.0123), Naive Bayes (*P* = 0.0084), and K-Nearest Neighbors (P = 0.0001). Furthermore, ET demonstrated comparable top-tier performance, with no significant difference from other tree-based models such as Random Forest (*P* = 0.2407) and LightGBM (P = 0.1017). Although Logistic Regression achieved a competitive mean AUC (0.930 ± 0.071) and high accuracy, its relatively large standard deviations (e.g., MCC SD = 0.288) suggested reduced stability across different data splits. In contrast, the ET model provided the most robust and consistent predictive performance.

**Fig 3:**
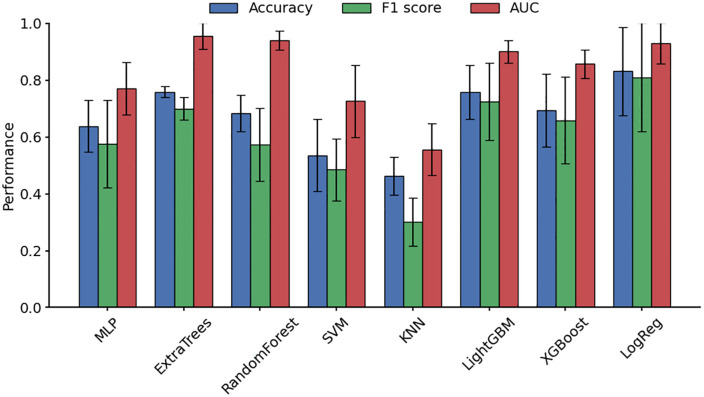
Performance comparison of nine conventional machine learning models. Bars represent mean performance (accuracy, F1 score, and AUC) across five-fold cross-validation. Error bars indicate standard deviation (SD).

Fig 3 presents grouped bar charts of accuracy, F1 score, and AUC for the nine conventional algorithms. Error bars represent standard deviations obtained from five-fold cross-validation, reflecting the variability of model performance across folds. The Extra Trees and Random Forest models showed relatively smaller standard deviations, indicating more consistent performance.

### Performance comparison of deep learning models

**Fig 4:**
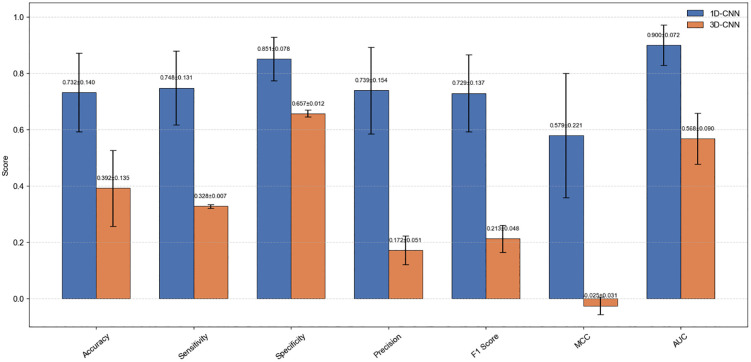
Comparison of 1D-CNN and 3D-CNN Performance Metrics.

Fig 4 compares the performance of the 1D-CNN and 3D-CNN models. Although the 3D-CNN integrated three-dimensional imaging, dose distribution, and oral anatomical structures, its predictive performance was inferior to that of the 1D-CNN under the current sample size, suggesting that high-dimensional architectures require further optimization when applied to small datasets.

### ROC curve analysis

**Fig 5:**
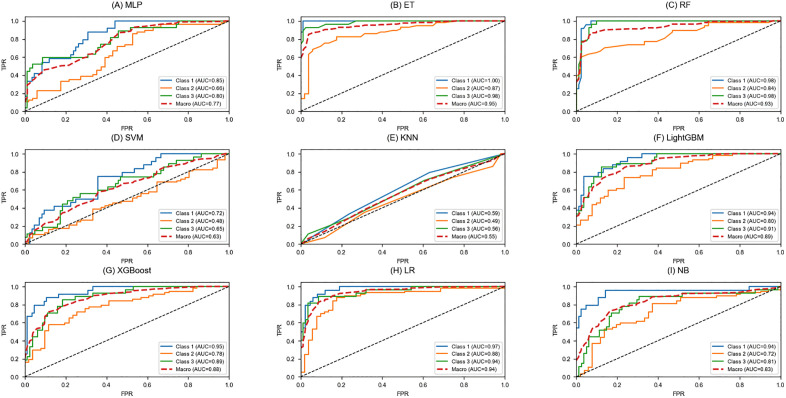
Comparison of ROC Curves Between Traditional Machine Learning Models.

**Fig 6:**
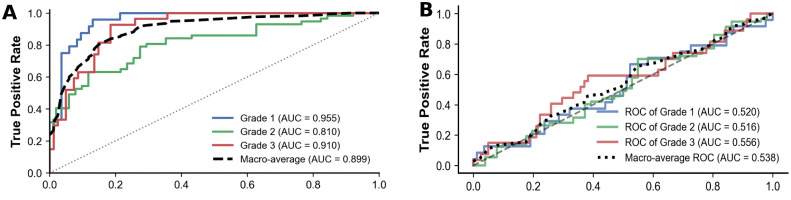
Comparison of ROC Curves Between Deep Learning Models.

Fig 5 presents the ROC curves of the traditional machine learning models, whereas Fig 6 illustrates the ROC curves of the deep learning models. LightGBM, XGBoost, logistic regression, Extra Trees and the 1D-CNN achieved larger areas under the curve, indicating more stable classification performance. In contrast, the curves of KNN, SVM, naïve Bayes, and the 3D-CNN were close to the diagonal, reflecting limited discriminative ability.

### Confusion Matrix Analysis

**Fig 7:**
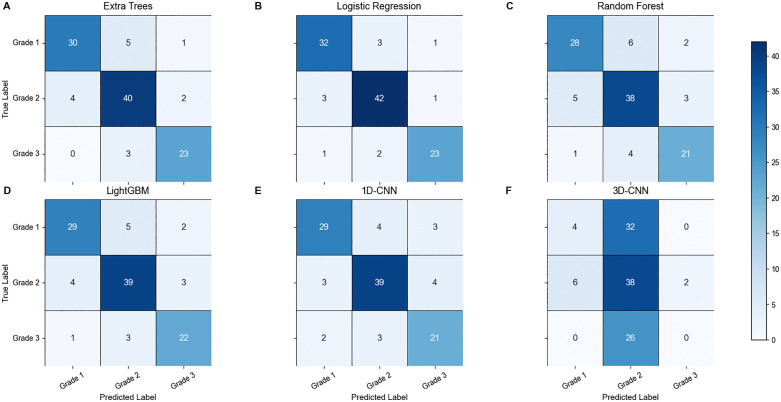
Confusion matrices of top-performing machine learning algorithms and representative deep learning models.

To facilitate an intuitive comparison of the classification performance among mainstream models, this study selected the best-performing algorithms in traditional machine learning (ET, LR, RF, LightGBM) and representative deep learning models (1D-CNN and 3D-CNN) to present their confusion matrices (Fig 7). The results of the remaining models are provided in Table 2.

As shown in Fig 7, the four leading machine learning models (ET, LR, RF, LightGBM) and the 1D-CNN exhibited relatively balanced recognition capabilities across all three severity grades. Their predictions were predominantly concentrated along the diagonal of the confusion matrices, indicating low misclassification rates and a strong ability to distinguish between adjacent toxicity grades. In contrast, the 3D-CNN model showed a substantial proportion of off-diagonal misclassifications,indicative of severe mode collapse, suggesting that its reliability could be further improved through data augmentation or structural optimization.

Beyond these representative models, Table 2 provides a comprehensive comparison of all algorithms under five-fold cross-validation. Among traditional machine learning methods, the ET model achieved the highest discriminative ability, reflected by the largest AUC value (0.956 ± 0.046). Meanwhile, LR demonstrated the most balanced and stable performance across accuracy, F1-score, and MCC, indicating strong robustness in multi-class classification. Other ensemble-based methods, including Random Forest, LightGBM, and XGBoost, also exhibited competitive performance, outperforming distance-based models such as KNN and probabilistic classifiers such as naïve Bayes. Although support vector machine (SVM) achieved moderate results, its overall stability was inferior to the best-performing models. Regarding deep learning models, the lightweight 1D-CNN achieved competitive performance (AUC: 0.900 ± 0.072), comparable to ensemble learning methods, whereas the 3D-CNN underperformed, likely due to insufficient sample size and high model complexity.

Overall, ET demonstrated the strongest discriminative capacity, while LR provided the most consistent and well-balanced classification performance. These findings suggest that both ensemble learning and appropriately regularized linear models remain highly effective strategies for structured clinical and dosimetric data in relatively small medical datasets.

### Model interpretability and modality contribution analysis

**Fig 8:**
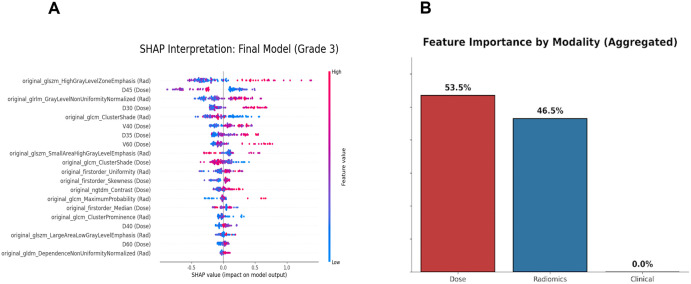
Model interpretability and modality contribution analysis. **(A)** SHAP summary plot displaying the top 20 features and their impact on model predictions. **(B)** Relative contribution of Dosiomics, Radiomics, and Clinical modalities to the multimodal model.

To improve model transparency and mitigate the “black box” concern, we employed the SHAP framework to interpret a representative high-performing model, LightGBM. Although the Extremely Randomized Trees model achieved the highest AUC, no statistically significant difference was observed between ET and LightGBM under five-fold cross-validation. Therefore, LightGBM was selected for interpretability analysis due to its strong predictive performance and suitability for feature contribution visualization. The SHAP summary plot (Fig 8A) reveals the top 20 most significant predictors, identifying a synergistic combination of dosimetric and radiomic features as the primary drivers for toxicity prediction.

Specifically, dosimetric parameters related to mid-to-high dose regions, including D_45_, D_30_, V_40_, D_35_, and V_60_, were ranked among the top predictors. This confirms that the volume of oral mucosa receiving 30–60 Gy is a critical determinant of mucositis severity. Crucially, radiomic features extracted from CT images played a distinct role. Features describing tissue heterogeneity and density patterns, such as High Gray Level Zone Emphasis (GLSZM), Gray Level Non-Uniformity Normalized (GLRLM), and Cluster Shade (GLCM), were identified as top contributors. High values of original_glszm_High Gray Leve lZone Emphasis (Rad) were strongly associated with model output, suggesting that inherent tissue density variations in the oral cavity modulate radiosensitivity. Furthermore, the analysis highlighted the importance of dosiomics (texture features of the dose map), such as Dose Skewness (First Order) and Dose Contrast (NGTDM). Unlike standard DVH metrics which only capture dose-volume histograms, these features characterize the spatial heterogeneity of the dose distribution, providing complementary information about dose “hotspots” and uniformity that standard metrics miss.

To explicitly evaluate the relative importance of different data modalities, we performed an aggregated contribution analysis (Fig 8B). The results indicated that the model’s predictive performance was predominantly driven by Dosiomics (53.5%) and Radiomics (46.5%). Notably, clinical characteristics showed a negligible contribution (0.0%) in the presence of these high-dimensional signatures. This suggests that the fusion of spatial dose distribution and tissue texture features provides a more comprehensive representation of the risk factors for severe RIOM than traditional clinical variables.

### Clinical utility and calibration

**Fig 9:**
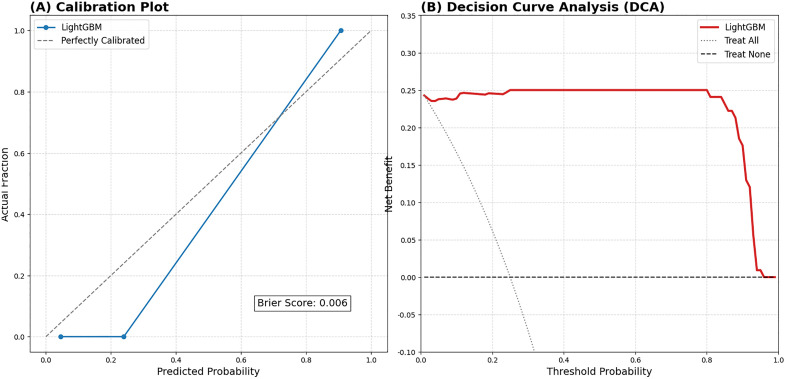
Clinical utility assessment. **(A)** Calibration curve with Brier score = 0.006. **(B)** Decision curve analysis (DCA) showing net clinical benefit.

The clinical reliability and practical value of the optimal model were assessed using calibration plots and decision curve analysis (Fig 9). The calibration plot (Fig 9A) showed a high degree of concordance between the predicted risk and actual observed outcomes, supported by a low Brier score of 0.006. Furthermore, the DCA (Fig 9B) demonstrated that the multimodal LightGBM model provided a superior net benefit across a wide range of threshold probabilities (approximately 5%–90%) compared to both the ‘treat-all’ and ‘treat-none’ strategies, confirming its high potential for clinical adoption.

## Discussion

In recent years, artificial intelligence (AI) has been increasingly applied in medicine due to its ability to process high-dimensional and non-linear data, with notable success in predicting radiotherapy-related complications such as pneumonitis and enteritis [8,12]. In this study, we developed a multimodal prediction model for radiation-induced oral mucositis (RIOM) and systematically compared nine machine learning and two deep learning algorithms. Our results showed that combining radiomics, dosiomics, DVH parameters, and clinical information markedly improved model accuracy and stability, underscoring its clinical utility.

Systematic reviews have noted that most existing oral mucositis (OM) prediction models rely on single-modality data [13]. Six studies have integrated clinical and DVH features, reporting internal validation AUCs of 0.62–0.81 [14–19]. Such performance may reflect the limited ability of single-modality models to capture complementary information across data types [20]. By contrast, our multimodal fusion approach achieved substantially better feature representation and predictive accuracy. For example, Hu et al. [21] reported an AUC of 0.883 and accuracy of 0.850 using multimodal multi-classifier fusion. In our rigorous 5-fold cross-validation, a top-performing cohort of algorithms emerged, comprising Extra Trees (ET), Logistic Regression (LR), Random Forest (RF), and LightGBM. Notably, there were no statistically significant differences in overall predictive performance among these four optimal models. Within this top tier, ET achieved the highest AUC (0.956) and LR reached the highest accuracy (0.832), both well above previously reported benchmarks. These findings highlight the inherent advantage of both tree-based ensemble learning (represented by ET, RF, and LightGBM) and appropriately regularized linear models (LR) for small-sample, high-dimensional, heterogeneous data, where multiple decision pathways enhance robustness and generalizability. The ensemble frameworks contribute by naturally handling complex interactions and resisting overfitting through multiple decision pathways, while the regularization strategies in LR improve resilience to noise. Collectively, these properties explain their superior predictive performance in this task. Furthermore, our modality-level contribution analysis (Fig 8B) explicitly quantifies the reliance of the optimal model on different data sources. The results indicate that Dosiomics (53.5%) and Radiomics (46.5%) are the primary drivers of the model’s predictive power. This finding aligns with recent studies suggesting that the spatial heterogeneity of dose distribution and inherent tissue texture patterns provide significantly more granular predictive information than macroscopic clinical factors [22,23]. Consistent with our observation, traditional clinical variables (e.g., TNM stage) often show minimal contribution when integrated with these high-dimensional quantitative signatures [24]. For deep learning models, the 1D-CNN outperformed the 3D-CNN under the current sample size (AUC: 0.900 vs. 0.568). The 1D-CNN relied on pre-extracted high-dimensional fused features, resulting in greater training stability. In contrast, the 3D-CNN directly incorporated CT images, dose distributions, and oral ROI structures, offering a framework closer to the clinical scenario. However, this architecture required larger sample sizes and greater computational resources, and given the limited training data and model complexity in this study, its performance was unstable. These findings are consistent with previous reports highlighting the vulnerability of deep models to training noise in small-sample medical tasks [25,26].

In addition, the use of standardized preprocessing, feature engineering, dimensionality reduction, and five-fold cross-validation effectively reduced overfitting and bias, thereby improving the robustness and generalizability of the predictive models. Among traditional machine learning approaches, ensemble-based models such as Extremely Randomized Trees (ET) and Random Forest (RF) demonstrated strong and stable discriminative performance, achieving high AUC values across folds. Notably, ET yielded the highest overall AUC, while Logistic Regression exhibited the best balance across accuracy, F1-score, and MCC. The tree-based ensemble models also offer relatively interpretable structures, making them suitable for feature importance analysis and clinical visualization. In contrast, distance-based and margin-based classifiers, including KNN and SVM, showed comparatively lower stability and overall performance. Although naïve Bayes achieved moderate results, its discriminative capacity remained inferior to the top-performing ensemble and linear models, suggesting limitations in handling multimodal high-dimensional medical data.

Our SHAP analysis provides biological and physical interpretability for the “black box” model (Fig 8A). Firstly, the prominence of DVH parameters such as D45, D30, and V40 aligns well with established clinical constraints. Previous studies have consistently identified mean dose and V30–V50 as key predictors for oral mucositis [23,24]. Our model rediscovered these physical laws solely from data, validating its reliability. Secondly, the inclusion of radiomic features (e.g., High Gray Level Zone Emphasis and GLRLM Non-Uniformity) among the top predictors provides additional insights into the biological basis of radiation-induced oral mucositis (RIOM). From a pathophysiological perspective, RIOM is initiated by DNA damage to basal epithelial cells, leading to oxidative stress, reactive oxygen species generation, and activation of pro-inflammatory cytokine cascades such as TNF-α and IL-1β [27]. Although causal relationships cannot be established, the predictive power of radiomic features suggests that pretreatment CT texture may reflect the baseline mucosal microenvironment. Increased heterogeneity may act as a macroscopic surrogate for subclinical inflammation, variations in mucosal thickness, or altered local vascularity, all of which may predispose tissues to an amplified inflammatory response following radiation exposure [28]. Thirdly, our study demonstrates the unique value of dosiomics. Features like Dose Skewness and Dose Contrast were ranked highly. The significance of these dosiomic features is consistent with the biological concept of ‘microscopic hotspots.’ While standard DVH parameters assume uniform dose distribution within a given volume, dosiomics captures spatial unevenness [29]. Highly skewed local dose distributions may cause disproportionate, focal depletion of epithelial stem cells, triggering a cascading, continuous ulcerative process that propagates to surrounding tissues, thereby exacerbating the clinical severity of mucositis. By capturing this spatial complexity and underlying biological damage mechanism, our multimodal model provides complementary predictive information beyond conventional DVH metrics.

While two patients might have identical Dmean, one might have a highly heterogeneous dose distribution (high skewness/contrast) with microscopic hotspots, leading to more severe local toxicity. By capturing this spatial complexity, our multimodal model surpasses traditional DVH-based prediction methods. In addition to discriminative performance, the clinical reliability and practical utility of our model were assessed using calibration curves and decision curve analysis (DCA) (Fig 9B). Unlike many previous studies that rely solely on ROC analysis, our inclusion of a calibration plot provides a measure of how closely the predicted probabilities agree with the actual observed risks. A low Brier score (0.006) indicates that the model is well-calibrated and offers precise risk estimates. Moreover, the DCA results demonstrate that the optimal multimodal model provides a significant net benefit across a broad range of threshold probabilities (approximately 5%–90%). This confirms that the model is not only a robust statistical tool but also possesses substantial practical value for assisting clinicians in identifying high-risk patients for early prophylactic interventions.

Despite the promising results, this study has several limitations. First, the sample size (n = 108) was relatively small, which constrained the performance of complex models like the 3D-CNN, although this cohort size is comparable to other recent pilot studies regarding AI-based mucositis prediction [30,31]. Second, the lack of an independent external validation cohort weakens the confidence in the model’s real-world performance across different institutions. Third, feature selection and hyperparameter optimization were mainly based on empirical approaches, without incorporating automated optimization frameworks such as AutoML.

To address these limitations, our team is currently engaged in continuous data accrual to expand the dataset. Furthermore, future work will focus on: (i) establishing a multicenter consortium to collect larger, heterogeneous datasets for external validation; (ii) optimizing deep learning architectures using automated machine learning (AutoML) techniques; and (iii) further refining the model’s interpretability to facilitate its integration into routine clinical practice.

In conclusion, the multimodal RIOM prediction model demonstrated favorable performance and strong clinical potential. These findings provide valuable insights for toxicity management in nasopharyngeal carcinoma radiotherapy and support the integration of AI technologies into precision radiotherapy.

## Supporting information

S1 Data Radiomic features dataset. (CSV)

S2 Data Dosiomic features dataset. (CSV)

S3 Data Clinical characteristics dataset. (CSV)

S4 Data Dose-volume histogram (DVH) parameters dataset. (XLSX)
